# Process evaluation with cost analysis of the Move for Life cluster randomised feasibility trial for inactive adults aged 50 years and older

**DOI:** 10.3389/fpubh.2025.1681089

**Published:** 2025-12-05

**Authors:** Enrique García Bengoechea, Ciaran Doyle, Amanda M. Clifford, Anna Hobbins, Liam Glynn, Andrew O’Regan, Chloe Forte, Andrew W. Murphy, Paddy Gillespie, Catherine B. Woods

**Affiliations:** 1 Physical Activity for Health Research Centre, Health Research Institute, Department of Physical Education and Sport Sciences, University of Limerick, Limerick, Ireland; 2 School of Allied Health, Ageing Research Centre, Health Research Institute, University of Limerick, Limerick, Ireland; 3 Health Economics & Policy Analysis Centre, J.E. Cairnes School of Business & Economics, University of Galway, Galway, Ireland; 4 School of Medicine, Health Research Institute, University of Limerick, Limerick, Ireland; 5 Centre for Public Health, Population Health Sciences, Bristol Medical School, University of Bristol, Bristol, United Kingdom; 6 HRB Primary Care Clinical Trials Network, Discipline of General Practice, University of Galway, Galway, Ireland

**Keywords:** healthy ageing, physical activity, program evaluation, mixed methods, intervention, community-based, implementation, health economics

## Abstract

**Background:**

An ageing population combined with a decline in physical activity with age have prompted calls for investment in physical activity programmes and services for older people. The Move for Life (MFL) intervention aims to augment existing community-based physical activity programmes for middle-to-older aged adults in Ireland using behaviour change strategies and peer support. The intervention was found to be feasible and has the potential to impact desired outcomes in intended ways. This study reports on the process evaluation of the MFL cluster randomised feasibility trial.

**Methods:**

The process evaluation used a sequential explanatory mixed methods design. Guided by the Medical Research Council framework, the evaluation sought to uncover links between implementation variables, mechanisms of impact and context of the intervention. The evaluation also addressed the dimensions of sustainability and cost of the intervention, as recommended in the RE-AIM framework.

**Results:**

While generally the trial reached the target audience, recruitment of males and individuals from hard-to-reach groups remained challenging. The evaluation revealed potential mechanisms underlying the effects of the intervention, notably improved attitudes toward physical activity, more positive appraisal of physical activity programmes and instructors, enhanced interpersonal interaction, greater social support, and higher use of individual and group behaviour change techniques. Regarding sustainability, MFL showed potential to strengthen the relationship between individuals and their Local Sports Partnership. The MFL intervention was associated with a statistically insignificant increase in mean cost relative to usual provision, and a statistically significant increase in mean cost relative to the control arm. While the evaluation supports the original MFL intervention logic model, it also suggests three major areas for improvement. These are the need to reconsider the use of the participant handbook for the delivery of behavioural skills as well as the number of behavioural change strategies included, particularly for shorter duration programme formats, and better integrate the training of peer mentors and physical activity programme instructors.

**Conclusion:**

The findings highlight the essential role of process evaluation to inform intervention design to facilitate optimal implementation and illustrate the value of considering several perspectives in understanding, collecting and using cost estimates from interventions.

**Trial registration:**

Identifier ISRCTN11235176, https://doi.org/10.1186/ISRCTN11235176.

## Introduction

As life expectancy around the world increases, the importance of healthy ageing and preventing avoidable falls and injuries is rapidly emerging as a critical health and economic issue that can be ameliorated by regular participation in physical activity (PA) (1). Despite the many well-documented physical, mental and social benefits of regular PA participation around a quarter of all adults worldwide – and over 40% of people aged over 70 years (38% of men and 48% of women) – do not do enough PA to maintain and improve their physical and mental health (2). In Ireland, only 34% of adults aged 55–65 years reported achieving National Physical Activity Guidelines and the percentage decreased to 29% in those aged 65–74 years, and 19% among those aged >75 years (3). Research has uncovered a number of factors for why PA declines with age, including a higher prevalence of long-term health conditions that may make being active more challenging and uncomfortable; misconceptions and inappropriate social norms about PA in older age; and lack of access to appropriate opportunities for being physically active (e.g., facilities, services and programmes) (1, 3, 4). The combination of an ageing population and growing prevalence of preventable noncommunicable diseases and prevalence of modifiable risk factors, particularly the decline in PA with age, underscores the need for investment in PA programmes and services for older people (1).

Between 2000 and 2009, with support from public funds administered via Sport Ireland, a network of 29 Local Sports Partnerships (LSPs) was established in Ireland to carry out a wide variety of actions aiming to increase sport and PA participation in local communities across the country (5). In 2020, over 340,000 people took part in sport and PA opportunities provided by LSPs (e.g., targeted programmes and events), of which over 7,000 participants took part in initiatives targeting older adults (5). The number of participants, combined with the strategic location of LSPs across Ireland, and the increase of public investments in LSPs to support their activities position LSPs suitably to contribute to address the high levels of inactivity among adults and older adults in Ireland.

To improve the prospect of adoption within, and scale-up across, the network of LSPs, the Move for Life (MFL) intervention was designed as an “augmentation” or enrichment of existing LSP programmes for adults and older adults instead of a new programme (6). MFL consists of three components that target behaviour change techniques theorized as the active ingredients for change: a training workshop for LSP PA instructors supported by a programme handbook, a training workshop for peer mentors and a programme handbook for MFL participants. Initial research provides evidence of feasibility and support for its positive impact on outcomes related to energy expenditure, body composition, and physical function over a period of 6 months (7). Furthermore, the intervention has shown potential to positively impact the psychosocial health and wellbeing of inactive adults aged 50+ years and change theorised mediators of physical activity, particularly when implemented as intended (8).

Process evaluation is valuable to inform intervention design and optimise implementation, enabling long-term sustainability and scale-up (9). Despite this, a systematic review of health promotion interventions in sport settings revealed that many process evaluations do not adhere to a process evaluation framework, and the ones that do use a variety of frameworks, highlighting the need for clarity in reporting to improve standardisation and integration (10). In addition, the perspectives of diverse stakeholders and use of multiple research methods are needed to understand the mechanisms that drive intervention outcomes (10). Likewise, a multi-stakeholder perspective has been advocated in understanding, collecting and using cost estimates from interventions, in a context in which cost and economic assessment and reporting are rare despite their essential role in implementing and sustaining evidence-based practices (11). In this study, we report on the findings from the process evaluation of the MFL trial, which we conducted according to guidance from the UK Medical Research Council (MRC) framework for the process evaluation of complex interventions (12), complemented with selected elements of the RE-AIM (Reach, Effectiveness, Adoption, Implementation, Maintenance) framework (13). Specifically, we used the former to understand (1) what was implemented and how; (2) mechanisms of impact; and (3) contextual aspects affecting implementation outcomes. As recommended in RE-AIM, we also considered sustainability of the intervention and provided a detailed cost analysis of the MFL trial.

## Methods

### The MFL trial study

The trial took place in pre-existing Local Sports Partnership (LSP) community sport and PA hubs in the Mid-West region of Ireland, developed under the National Physical Activity Plan and whose purpose is to increase physical activity, especially among disadvantaged and marginalised groups. A total of eight hubs across counties Clare (*n* = 4) and Limerick (*n* = 4) were recruited. Hub inclusion criteria required professional expertise to run four nationally approved PA programmes suitable for inactive middle-aged and older adults. These were Men on the Move (an evidence-based mixed sport programme for men; 12 weeks, 2 sessions/week), Women on Wheels/Bike for Life (a ‘Get Ireland Cycling’ cycling programme; 10 weeks, 1 session/week), Go for Life (an indoor mixed games programme developed by ‘Age and Opportunity,’ the national organization working to enable the best quality of life for Irish adults as they age; 8 weeks, 1 session/week) and Get Ireland Walking (an outdoor community walking programme; 10 weeks, 1 session/week). In total, 32 freely accessible PA programmes were implemented over the trial period.

A cluster design was used to overcome spillover effects. LSP hubs were the units of randomisation (clusters) and participants within these hubs (units of analysis) were randomised to one of three arms, (i) MFL intervention group (MFL; existing PA programmes plus MFL augmentation, 3 hubs); (ii) the usual provision (UP; existing PA programmes delivered as normal, 3 hubs); and (iii) the control group (CON; PA information only, 2 hubs). CON individuals were invited to participate in the PA programmes after the trial. Each hub was geographically separated to reduce contamination, and clusters were stratified as rural or urban.

The MFL intervention consists of three components: a workshop for LSP instructors (supported by a handbook), a workshop for peer mentors, and a handbook for participants. It has been described in detail elsewhere (6). In brief, MFL aimed to enhance the impact of established national PA programmes by augmenting the professional model with a multimodal intervention. LSP instructors, trained in the delivery of the PA programmes mentioned above, attended two 3-h workshops on behavioural theory and integrating behaviour change techniques into their programmes. These techniques focused on developing cognitive and behavioural skills, promoting social support, and fostering group cohesion. Additionally, instructors recruited suitable volunteers as peer-mentors, who completed a 3-h workshop to enhance PA programme peer support, contribute to group sustainability and identify useful resources available for participants. MFL handbooks supported the training with a delivery guide for instructors and a participant handbook including behaviour change information and individual and group tasks.

### Design of the process evaluation

The process evaluation adopted a sequential explanatory mixed methods design where qualitative data complemented and contributed to the interpretation of quantitative data (14). Quantitative and qualitative data were integrated in the results section. The evaluation drew upon the logic model of the MFL intervention (6) and was guided by the Medical Research Council (MRC) framework for the evaluation of complex interventions (12). In line with this framework, the evaluation sought to shed light on the links between implementation variables, mechanisms of impact and context of the intervention. In addition, the evaluation addressed the related dimensions of sustainability, adaptations to and cost of the intervention, as recommended by the RE-AIM framework (13).

### Data collection

Data collection took place from May 2018 to June 2019. All experimental protocols were approved by University of Limerick, Faculty of Education and Health Sciences Research Ethics Committee (Registration No. 2018_02_15_EHS; 09 April 2018) and were carried out in accordance with the Declaration of Helsinki for research involving human subjects. All participants provided written informed consent. All participants provided written informed consent to take part in the study, including publication of any potentially indirectly identifiable data in this article.

#### Instructor evaluation of training workshop

Instructors completed pre- and post- MFL workshop questionnaires. The self-report questionnaire consisted of eight questions asking the respondents to rate their competence in delivering behaviour change strategies in PA settings. Responses were given on a Likert scale ranging from 0–10, with 10 being high.

#### T0, T1, T2 programme questionnaire

Participants completed questionnaires at baseline (T0), post intervention (T1, at 8, 10 or 12- week), and 6-month follow up (T2). These captured demographic data (age, gender, principal status, marital status, education, and presence of comorbidities). Data was also collected on hypothesized mediators of PA (attitude, subjective norms, behavioural control, intention, self-efficacy, decisional balance, and relatedness to others in physical activity) using validated measures (15–19).

#### T1, T2 process evaluation questionnaire

All participants were asked to complete self-report process evaluation questionnaires post-intervention and at 6-month follow-up. Participants rated their level of agreement using a 5-point Likert scale to a list of statements related to Move for Life and responded to open-ended questions related to their experience of the Move for Life intervention.

Participants completed process evaluation questionnaires post-intervention and at 6-month follow-up. They rated statements such as “the session length was appropriate” or “I contacted peer mentors,” using a 5-point Likert scale for agreement (strongly agree, agree, no preference, disagree, strongly disagree) or frequency (very frequently; frequently; occasionally; rarely; never) depending on the question asked. Open-ended questions also captured feedback, e.g., suggestions for improving venue or timing.

#### Participant attendance sheets

Instructors maintained participant attendance logs for each session. This was collected by the MFL facilitator at programme end.

#### Instructor fidelity checklists

Each intervention group instructor used handbook checklists to document weekly delivery of MFL intervention components, returning them to the facilitator at programme end.

#### WhatsApp log

Intervention instructors communicated with the MFL facilitator via WhatsApp for support and to confirm delivery of intervention components as designed. Logs also provided insight into instructor experiences.

#### Instructor, participant, peer mentor, and MFL facilitator interviews

Semi-structured interviews were conducted with instructors (UP: *n* = 6, MFL: *n* = 11), peer mentors (*n* = 15), the MFL facilitator (*n* = 1) and participants (UP: *n* = 23, MFL *n* = 17). The interview guide used is accessible in Supplementary material S1. Interviews were conducted face-to-face where possible and via telephone. All interviews were audio recorded and transcribed verbatim.

### Data analysis

#### Instructor evaluation of training workshop

Pre and post workshop questionnaire scores were input into IBM SPSS Statistics Version 25. Mean scores were calculated and presented for each item.

#### T0, T1, T2 programme questionnaire

Descriptive analyses were conducted on demographics and hypothesized PA mediators. A mediation analysis extended a previously reported multivariate mixed-effects regression model (7) examining MFL’s effect on self-report compliance with PA guidelines. According to classic criteria (20), we assumed a mediation effect when (1) the exposure (MFL intervention) significantly affected the mediator (e.g., change in attitudes to physical activity), (2) the exposure significantly affected the outcome (change in compliance with guidelines) in the absence of the mediator, (3) the mediator had a significant unique effect on the outcome, and (4) the effect of the exposure on the outcome got smaller upon the addition of the mediator to the model.

#### T1, T2 process evaluation questionnaire

Responses for agreement were collapsed into two categories (strongly disagree/disagree/neutral = disagree, strongly agree/agree = agree) for descriptive statistics. Group differences (intervention vs. usual provision) were explored using binary logistic regressions to estimate odds ratios with 95% confidence intervals. Paired samples *t* tests were used to investigate any difference within each group at different time points.

#### Participant attendance sheets

Attendance was calculated for each participant using a percentage based on actual number of sessions attended out of total number of sessions delivered.

#### Instructor fidelity checklist

Session compliance was based on the proportion of required components delivered. Overall compliance was averaged across weeks and categorized (low, medium, high) using previously recommended procedures (21).

#### Whatsapp log

The WhatsApp communication log was downloaded into a word document. WhatsApp messages were reviewed by two members of the research team to quantify unique instructor-facilitator interactions, including those initiated by instructors. Data are presented as counts.

#### Qualitative data from interviews and open-ended responses in process evaluation questionnaire

Inductive content analysis was used to identify patterns and make sense of the available qualitative data. The coding process involved two phases; initial coding and focused coding (22). Initial coding followed an incident-to-incident approach, whereby each incident in the transcripts was coded with phrases or words which were used by the participants. In the second phase of coding, focused coding, categories and sub-categories were constructed. Initial codes and the categories and sub-categories were contrasted through a constant comparison approach in a more selective and conceptual manner. The research framework and questions were drawn on in this phase to provide context to the focused codes and subsequent categories. Pertinent quotes are used throughout to contextualize quantitative findings.

#### Follow up phone calls (drop out)

Reasons for dropout were coded and reported as descriptive statistics.

### Cost analysis

The cost analysis covered the 6-month trial follow-up period and was expressed in Euros (€) in 2019 prices. Given the length of follow-up, costs were not discounted. Two resource components were included in the cost analysis, which reflect the perspective of the service provider (LSPs) and the healthcare system (Health Service executive), respectively. The first was the cost of organising, implementing and running the MFL intervention, the usual provision and the control arms. This included the resources relating to the operation of four LSP PA programmes: Men on the Move, Women on Wheels/Bike for Life, Go for Life and Get Ireland Walking. In addition, for the MFL intervention arm, the additional costs relating to the behavioural augmentation and peer mentor support model were included. In particular, the resource requirements captured included those relating to professional personnel time input for training sessions for instructors and peer mentors, venue and equipment rental, informational consumables and materials, travel costs, printing, postage and administration. Second, costs relating to the use of primary and secondary healthcare services over the course of the trial period were estimated. This included the costs of general practitioner (GP), practice nurse, outpatient services, inpatient nights and accident and emergency (A&E) visits.

Resource use data was captured from study accounts and participant questionnaires completed at baseline, 3 months and 6 months. Given the length of follow up, costs were not discounted. A vector of unit costs was sourced and applied to calculate the cost associated with each resource activity. Unit cost estimates for the four PA programmes were recorded in the study accounts and unit cost estimates for each healthcare service were obtained from national data sources and, where necessary, were transformed to Euros (€) in 2019 prices using appropriate indices (23, 24).

For the statistical analysis, a total cost variable was constructed for the purposes of undertaking incremental cost analysis, which comprised of the sum of the costs of organising, implementing and running the PA programmes and the costs relating to the use of primary and secondary healthcare services. Estimation of incremental costs at follow-up was undertaken using a multilevel generalized estimating equations regression model, assuming a Gaussian variance function, controlling for treatment arm, baseline cost, and clustering by hub where programmes were offered.

The impact of varying the MFL intervention programme costs by plus and minus 10 and 50% was considered in sensitivity analyses, as was a univariate regression analysis, and a multivariate regression analysis of the MFL intervention costs only (that is, excluding other healthcare costs).

## Results

Based on the MRC framework, results of the MFL trial process evaluation are divided into three domains: (1) intervention implementation, (2) mechanisms of impact, and (3) context of implementation. In addition, results from the cost analysis are presented.

### Implementation of the Move for Life programme: what was delivered and how?

Complex interventions, such as MFL, may have limited effects on targeted outcomes and this may be related to either weakness in intervention design, or because the intervention was not properly implemented (12). To understand what was implemented, this section reports on the characteristic of the study participants (reach); number of sessions delivered by the instructors (dose delivered); participant attendance at those sessions (dose received); and the extent to which instructors delivered the intervention as planned (fidelity).

#### Characteristics of trial participants (reach)

Over 700 (*n* = 733) participants registered to become part of the MFL trial. Most (98%, *n* = 724) consented and completed baseline measures. The inclusion criteria for MFL specified ≥50 years and inactive, i.e., not meeting the PA guidelines. In total, 18% (*n* = 132) were excluded from the research due to age or activity status. A comparison between included and excluded individuals found that those excluded from MFL research were younger, more active and that proportionally more males were excluded than females from the study (Supplementary Table S1). Participant sociodemographic characteristics are shown in Table 1. The average age of the study sample was 63.06 years, and the range was 50 to 91 years. The majority of the study sample were female (80.4%). Participants in the usual provision group were significantly older than in other groups. No statistically significant difference was found in gender, for medical card holders, for having private health insurance, nor in education level across trial arms. Participants in the control arm were more likely to be separated or divorced than those in the intervention or usual provision arms. Participants in the MFL study were more likely to be working and have gained a tertiary qualification compared to respondents in the nationally representative Healthy and Positive Ageing Initiative (HaPAI) survey (25) (Supplementary Table S2). Similar prevalences of retirement and marriage were reported by respondents in both studies. The sample in the current study reported higher education attainment and greater likelihood to be working than HaPAI respondents.

**Table 1:** Characteristics of MFL study participants by trial arm (*n* = 601).

	Intervention	Usual provision	Control	*p* value
	Mean (SD)	Mean (SD)	Mean (SD)	
	(*N* = 189)	(*N* = 269)	(*N* = 143)	
Age (years)	61.86 (7.98)	64.22 (8.51)	62.50 (7.37)	0.006
Male /female (mean)	62.3/61.8	65.4/63.8	61.4/62.7	
	*N* (%)	*N* (%)	*N* (%)	
Gender				
Male	29 (15.3%)	58 (21.6%)	31 (21.7%)	0.2
Female	160 (84.7%)	211 (78.4%)	112 (78.3%)	
Medical card (yes)	56 (30.8%)	102 (38.9%)	45 (31.9%)	0.15
Private health insurance (yes)	147 (79.9%)	213 (81.0%)	116 (82.3%)	0.864
Highest level of education				
Primary or no formal training	16 (8.7%)	22 (8.4%)	6 (4.3%)	0.162
Lower secondary	30 (16.3%)	37 (14.1%)	23 (16.3%)	
Upper secondary	50 (27.2%)	57 (21.8%)	32 (22.7%)	
Post-secondary non tertiary	6 (3.3%)	17 (6.5%)	3 (2.1%)	
Non-degree	35 (19.0%)	65 (24.8%)	44 (31.2%)	
Degree or higher	47 (25.5%)	64 (24.4%)	33 (23.4%)	
Marital status				
Married/living with partner	128 (69.2%)	170 (64.6%)	87 (61.7%)	0.044
Widowed	24 (13.0%)	39 (14.8%)	24 (17.0%)	
Separated/divorced	19 (10.3%)	22 (8.4%)	23 (16.3%)	
Single/never married	14 (7.6%)	32 (12.2%)	7 (5.0%)	

#### Number of sessions delivered by the instructors (dose delivered)

In total, 10 groups participated and 97% of intended sessions were delivered across the intervention arm. Two groups did not complete all their sessions as stipulated. One group ceased 5 weeks into an eight-week programme due to low participant attendance and the other ceased 7 weeks into an eight-week programme. In total, 11 groups participated in the usual provision arm. All these groups completed their sessions as prescribed by their programme, thus 100% of sessions were delivered.

#### Participant attendance at sessions (dose received)

Of the 405 participants who were randomised to either intervention or usual provision treatments, 292 (72.1%) attended at least one session. Average attendance was higher in the intervention group (63.6%) compared to the usual provision group (59.5%). Notably though, 113 (27.9%) participants who registered to participate in the study and were allocated to a programme never attended a session, which was more prevalent in the usual provision arm (*n* = 80; 33.2%) compared to the intervention arm (*n* = 33; 20.1%).

#### Extent to which instructors delivered the intervention as intended (fidelity)

Average compliance with the intervention was calculated for all groups who returned fidelity checklists filled by instructors (10 groups out of 11). According to fidelity checklists, 508 out of 662 (76.7%) of MFL intervention components were delivered by the instructors. In addition to *what* was delivered, it is important to report on *how* the delivery is achieved as this provides information on how the intervention can be replicated (12). The following sections report on recruitment procedures for instructors, peer mentors and participants, as well as instructor and peer mentors’ training and support and their responses to the training they received.

#### Recruitment of instructors

The LSPs were tasked with providing instructors to attend training and deliver the Move for Life programme as well as additional instructors to deliver the usual provision classes. In total, 23 instructors were recruited to teach the LSP PA programmes to the intervention and usual provision hubs.

The qualifications and experience of the MFL instructors selected by the LSPs to deliver the programme was very varied. While this may have made recruitment easier, it also created some challenges. A minimum expectation of expertise and experience and appropriate management of instructors during the programme was recommended in an interview by the MFL facilitator:

“I think their qualifications are very different. One of the individuals that came [to the instructor training], I think she was a cycling leader and, she never came back. Maybe she got a little bit overwhelmed by the delivery of the programme and things like that.” (MFL facilitator).

#### Training and support for instructors

Prior to delivering the MFL programme, all instructors were required to attend training sessions with the MFL research team. Instructors were asked to complete a workshop evaluation pre- and post-workshop to assess their level of confidence regarding skills deemed important for delivery of the MFL intervention. Responses were recorded on a 10-point Likert scale. Instructors reported a notable increase in confidence to deliver the eight components after completion of the training workshop, particularly for implementing social support strategies and using a way of communication that gives choice and initiative to the participants (Table 2).

**Table 2:** Instructor confidence to deliver intervention strategies before and after training (*n* = 14).

	Before training mean	After training mean
Teaching behavioural skills to motivate participants	5.6	7.9
Implementing social support strategies	6.1	8.5
Fostering group integration and cohesion	5.8	8.0
Demonstrating and explaining tasks	6.5	8.1
Providing specific feedback and encouragement to enhance learning and motivation	6.4	8.4
Implementing strategies that foster intrinsic motivation	6.5	8.3
Using a way of communication that gives choice and initiative to the participants	6.1	8.5
Working with and supporting assistants to achieve long term sustainability of the programme	6.4	8.4

Range 0–10.

Communication via WhatsApp was maintained between the instructors and MFL facilitator throughout the programme delivery phase to ensure instructors were supported. In total, there were 42 unique interactions between the MFL facilitator and MFL instructors using WhatsApp. Of these, 52.4% were initiated by the instructor. Primary reasons for contact predominantly related to fidelity checklists, participant attendance, and peer mentor training.

Instructors were asked to report on their experience of the MFL training during interviews. They commented on aspects of the training they found beneficial, such as the psychological component to motivate individuals and the group, the opportunity to share ideas and give and receive feedback and gaining an increased understanding of how to teach skills:

“As I said, I am involved in coaching, but for those who have not a great deal of coaching, then it would be definitely beneficial for them, and like I said people gave ideas, people gave, like I gave feedback from what I had done in classes, people gave other feedback… So, it helped the whole group to understand how to teach and coach and how to keep the group going.” (MFL intervention instructor).

Main recommendations for improvement of the MFL instructor training were inclusion of more hands-on and interactive activities. Some concerns were also expressed about the relevance of the instructor handbook in practical terms.

#### Recruitment and training of MFL intervention peer mentors

Three peer mentoring training workshops were run as part of the MFL study. The first MFL peer mentor programme was felt to be the least successful, in part, due to problems recruiting peer mentors prior to the beginning of the MFL programme, as initially contemplated. Lessons learned were used to adapt and improve the recruitment process for subsequent programmes, so that peer mentors could be recruited, and trained, once the MFL programme had started. Twenty-seven MFL participants (78% female) completed this training and became MFL peer mentors. Peer mentors had a higher average class attendance than non-peer mentors (81% vs. 71%). The overall retention rate for peer mentors was higher than for regular participants at 6 months post baseline (76% vs. 63% for the whole sample).

The instructors recruited the peer mentors through different means which included choosing individuals who volunteered and through a voting system. The instructors stressed the importance of picking the right peer mentor and the effect it can have on the group. One instructor commented on the positive effect the voting system had on the chosen individual and the group:

“The lady that we chose in the end that they voted on, she was extremely quiet…but when she was put in that role, she just, kind of, she took charge. So, I would have never seen that in her. I suppose letting the group decide for themselves, they have a better feel of who they want rather than me picking it out.” (MFL intervention instructor).

Views on the peer mentor training differed. While some peer mentors found it was useful, easy to understand and relevant to practice, others felt the training included too many things given the time allocated and highlighted the need for additional training as they did not feel fully prepared to be a peer mentor. To avoid some of the issues raised, the MFL facilitator recommended that the instructors attend some or all the peer mentor training:

“I think it should be 100% compulsory that the instructor goes to training with the peer mentors - even to build up that bond between them… get the instructor to write out a few things that they find difficult to deal with and the peer mentor might go, well I’d love to try that or something like that. So that connection was definitely missing.” (MFL facilitator).

This sentiment was echoed by a peer mentor, when referring to what she perceived as a lack of communication with the instructor and shortcomings with the training, which hindered her role in the MFL programme:

“When I came to the training, I thought it would interest me but then when I went back the next night to the walking, I felt, you know, we are back to where we started again and, you know, we just were not being mentored [by the instructor]. I feel and I thought that should have started, you know, during the training… encouragement, motivation [was missing]… from our trainer or from the leader, whatever” (peer mentor).

In contrast, some peer mentors commented how their instructor taught them different approaches and skills to use within the programme, while instructors seemed to appreciate the value peer mentors could bring both during and after the programme:

“So, what the peer mentor would do is she count down for me or she’d step in when I was showing someone how to do something correctly or fixing them or she would step in for me and count down or you know watch the time. It was great in that sense it took the pressure off me” (intervention instructor).

“The whole thing about the legacy, the whole thing about improving people’s health is that they have been doing it for 8 weeks, it’s can they do it for 52 weeks a year? So, it’s crucial to have someone there left behind who will still help facilitate that” (intervention instructor).

#### Recruitment of participants

Recruitment was multifaceted via radio interviews, press releases in national and local papers, notices in newsletters and community notes, notices read out at religious services, presentations to local groups (e.g., Irish Country Woman’s Association, Men’s Sheds etc.), presentations at General Practitioners meetings, notices to library groups, text messages via community alerts, posters and leaflet drops at shops, resource centres, bingo groups etc., and social media posts via various targeted interest groups. Most participants reported that they heard about Move for Life through word of mouth (40.3%). Of those who heard through word of mouth, 58.3% heard from a family member or friend, 10.4% heard from others, and 5.7% heard through their General Practitioner.

### Mechanisms of impact: how does the delivered intervention produce change?

In line with the MRC framework, an analysis of mediators of change, and relevant mechanisms related to participant response to and interaction with the intervention are reported below.

### Mediators of behaviour change

Average daily minutes of device-measured MVPA and self-reported compliance with PA guidelines were the primary outcomes of the MFL trial. Even though MVPA decreased over the study period for the whole sample, the decline among control group participants was significantly larger than among Move for Life and usual provision participants, which were not significantly different from each other (7). Therefore, we conducted an exploratory mediation analysis on self-reported compliance with PA guidelines, which was significantly higher over time among MFL participants compared to usual provision participants. These, in turn, reported significantly higher compliance over time than controls (7).

An analysis accounting for implementation fidelity suggested that the association over time between the MFL intervention and compliance with PA guidelines was partially explained by changes in attitudes toward physical activity, but only in the ‘high fidelity’ MFL intervention group. According to established criteria (20), this was evident in that (1) the exposure (MFL intervention) significantly affected the mediator (change in attitudes) (*B* = 0.11; 95% CI = 0.01, 0.20), (2) the exposure significantly affected the outcome (change in compliance with guidelines) in the absence of the mediator (OR = 34.39, 95% CI = 2.92, 405.16), (3) the mediator had a significant unique effect on the outcome (OR = 1.45, 95% CI = 1.02, 2.06), and (4) the effect of the exposure on the outcome got smaller upon the addition of the mediator to the model (from OR = 34.39, 95% CI = 2.92, 405.16 to OR = 29.86, 95% CI = 2.62, 339.88), after controlling for relevant confounders.

Likewise, baseline levels of self-efficacy to overcome barriers to PA had a unique significant effect on the outcome (OR = 1.80, 95% CI = 1.45, 2.24). This variable met the remaining criteria to be considered a mediator of the association between the intervention and change in compliance with PA guidelines in the MFL high fidelity group after accounting for relevant covariates. This was also the case when the three original groups (i.e., MFL intervention, usual provision, control, with no consideration of implementation fidelity in the former) were used in the analysis (OR = 1.92, 95% CI = 1.56, 2.36). In such analysis, although there was no clear evidence that change in relatedness to others met the first criterion to be considered a mediator, it was the only hypothesized psychosocial determinant that remained significant in the final model (OR = 1.14, 95% CI = 1.01, 1.30) and somewhat reduced the effects of the intervention, even if marginally (from OR = 13.00, 95% CI = 3.27, 51.51 to OR = 12.74, 95% CI = 3.02, 53.71).

In addition to the mediational analysis, findings from the process evaluation questionnaire, shed light on the mechanisms of impact of the MFL intervention, specifically in terms of participant response to and interaction with the intervention.

#### Participant reasons for joining or dropping out of the programme

During the process evaluation questionnaire, participants were asked an open-ended question about the reason why they joined Move for Life. The main reasons offered by intervention and usual provision participants are detailed in Supplementary Figure S1. Reasons for joining were varied but predominantly related to health, fitness and social considerations. Both groups reported that getting active was the primary reason for joining the programme, with meeting new people cited often.

Follow up phone calls were made with all participants who had dropped out of the study. Reasons for post-intervention (T1) dropout are pertinent as this was the testing period immediately after the programme ended. For those who offered a reason, medical reasons (5.9%) was the most commonly cited response, followed by other commitments (e.g., family) (Supplementary Table S3).

#### Participant overall views and experience of the LSP PA programme

Participants were asked to rate their agreement with items relating to their experience with the corresponding LSP PA programme (Supplementary Table S4). While controlling for age differences, the intervention group participants were significantly more likely to find their programme interesting (OR = 3.04; 95% CI = 1.06–8.76; *p* = 0.039); they felt the programme was worth the time they invested in it (OR = 3.76; 95% CI = 1.33–10.62; *p* = 0.012); they were more likely to report that their group enjoyed the programme (OR = 2.56; 95% CI = 1.16–5.65; *p* = 0.02); and were also more likely to recommend the programme to a friend (OR = 3.52; 95% CI = 1.11–11.12; *p* = 0.032) than usual provision participants.

The most valuable aspect of the MFL programme according to the participants was the social interaction. Throughout the interviews, participants consistently referred to meeting new people, ‘chatting’ to other participants and being part of a group as aspects which they found most enjoyable. The social aspect of doing PA with others emerge as a motive to continue the activity or do new physical activities and some appreciated how they now have a group of people that they can now contact to do PA with:

“Well, I met two people… who I now, we walk another night in the week as well which is really nice for me because… I would walk around from my house, but it was by myself whereas now I’ve got two people that I can text and say, ‘I’m going for a walk in [location] tonight, are you able to come or not?’ so that’s been a real benefit” (intervention participant).

For both intervention and usual provision groups, the activity was reported as one of the most enjoyable aspects of the programme, often in combination with the social aspect of participation (see Supplementary Figure S2). The intervention group were more likely to cite group exercise as the most enjoyed aspect of their programme. Open question responses highlighted aspects of the intervention, including group integration and goal setting:

“The actual group walks, the chat made the time fly” (intervention participant).

“Working as a group with us all having the same goal” (intervention participant).

Other participants enjoyed the structure programmes provided and the safety aspect of participating in a group:

“It was structured, yes, that’s what I liked about it, and you felt safe walking in a group. Sometimes you are not safe out at night on your own walking” (usual provision participant).

The least enjoyed aspect reported by both groups was the weather, which will be discussed in a section on context of the intervention below. Logistics associated with travelling to distant venues, research burden (e.g., lengthy questionnaires) and, as illustrated by the quote below, issues related to other participants (e.g., different ability levels) were also commonly mentioned (see Supplementary Figure S3):

“I found it [the programme] enjoyable but…it wasn’t really a beginners walk for life programme I felt, I’m a beginner…I found it very hard to keep up with everyone… I found I missed out on a lot of the talks because by the time I came around a lot of the conversation was over” (intervention participant).

Issues about the duration of the programme were also raised by some MFL participants and the MFL facilitator. In particular, the 8-week programme was felt to be too short, with a lack of time to absorb and put new behavioural skills learned into practice. Despite this, goal setting, one of the main behavioural skills focused on in the MFL intervention, proved very helpful for some participants:

“Definitely, the goals and the steps of goals on the way were hugely beneficial and very helpful” (intervention participant).

#### Interaction between participants and instructors

Most participants rated their instructors very positively, with scores on 8 out of 10 items in the high 90s (Supplementary Table S5). This is positive reinforcement of the instructional role of the LSP personnel. This also highlights the challenging position for any programme to improve further on these scores. Yet, despite these exceptional grades the MFL intervention received significantly more positive feedback than usual provision on mixed ability teaching (OR = 2.06; 95% CI = 1.07–3.96, *p* = 0.030) and providing participants with more choices on how to perform the activities (OR = 3.03; 95% CI = 1.56–5.88, *p* = 0.001). Furthermore, at T2 follow up, participants were also asked if they had contacted their instructor since the MFL programme had finished. Significantly more of the intervention group, in comparison to the control group, reported that they had contacted the instructor frequently or very frequently (OR = 6.95; 95% CI = 2.55–18.93, *p* = 0.001) since the conclusion of the structured exercise session. This links to the programme sustainability considerations below.

Responses to the open questions were mostly positive and related to helpfulness of instructor, provision of motivation and encouragement, and the personality of the instructor:

“Instructors were brilliant - they kept us going and made it enjoyable” (intervention participant).

“They were very encouraging and good humoured, very positive” (Usual provision participant).

There was also some constructive feedback offered which predominantly related to catering for mixed abilities, communication, and coping with changes in instructors:

“Unfortunately, the first tutor [instructor] had to leave after week 7 and had built up a good rapport with the group and we had three other tutors for the remainder. It would great if you had the same tutor” (Usual provision participant).

Often, instructors were supportive of the different participant abilities, as one participant recalled:

“No [my ability did not affect my enjoyment of the programme] because I worked within my own limits because I said it to the instructor…and I do not feel embarrassed that I’m not able to do everything that everybody else is able to do and the one thing he did give me, which was brilliant, he gave me, which I’ve started doing at home, little exercises sitting in the chair” (intervention participant).

This, however, was not always the case, and led participants from both groups to express a preference for broader choice of activity and the need for scheduling programmes based on ability level:

“Very good and knowledgeable but very focused on cycling fast and part of the group- not sure that they took all the needs of the group into consideration- we went at the pace of the fastest person” (intervention participant).

#### Participants’ response to MFL workbook and materials

As can be seen in Supplementary Table S6, many participants agreed that the workbook for MFL was appealing (76%), and weekly homework tasks were easy to understand (77.8%). However, fewer participants agreed that they engaged with the ‘homework tasks’ every week (54.5%), with some indication in the responses to open questions that the MFL handbook might not have been followed by all instructors as was intended:

“Maybe we could have looked at the workbook before starting the walks - not just given it as a handout for yourself to use it” (intervention participant).

Engagement with the handbook was varied. While for some it was *“a bit like school,”* others discussed how the handbook should be more educational with more focus on exercises with pictures and demonstrations of how these could be done at home. Similarly, while some instructors found the handbook helpful and referred to it more throughout the programme, others did not recognise the value in doing the homework:

“The programme is a very good idea, but I do not know, it’s very hard to motivate over fifty-year olds and then giving them homework in the process. I think that’s the turn off… I think the homework was like, like I’m 32 and If I go for my fitness class and somebody will, like after the class, “you have to do this”, I would be like okay good luck with that” (intervention instructor).

Finally, others commented how bringing the handbook was impractical due to the nature of the activity (e.g., cycling). One instructor discussed how he got the group to do the homework as a group to overcome the issue of getting participants to complete the homework:

“I mean they are adults, and you know, they go home, and they have got responsibilities and everything so… That it was stressful trying to do it at home. So, I mean after the second week I said it to them I said all right you know we can do it together, which I think was better because we got more discussed and more thought went into it” (intervention instructor).

Participants were asked open-ended questions about how to improve the MFL intervention. Responses were primarily related to intervention materials, communication, setting, and activity (see Supplementary Figure S4). The usual provision group asked for more handouts regarding physical activity and its benefits. This was not requested by the intervention group and may be because they already had the MFL handbook.

#### Participants’ response to behavioural change techniques used in MFL

The MFL intervention was designed to enhance the use of BCTs by the participants, usual provision did not receive this augmentation, so these process evaluation questions addressed this topic. Use of key behavioural change techniques was assessed at T1 and T2 (Supplementary Table S7). At T1 more of the intervention group, in relation to the usual provision, reported enjoying learning about different strategies to keep physically active (OR = 2.62; 95% CI = 1.31–5.27, *p* = 0.006); setting personal goals to increase PA (OR = 1.07; 95% CI = 1.03–1.11, *p* = 0.001); and setting a clear group goal to remain active (OR = 2.81; 95% CI = 1.64–4.81, *p* = 0.001).

At T2, as compared to the usual provision, the intervention group reported considering the personal benefits of becoming more active (OR = 2.4; CI = 1.01–5.7, *p* = 0.047); discussing the personal barriers to becoming more active (OR = 2.1; CI = 1.09–4.04, *p* = 0.026); and to clearly setting a group goal to help them remain active after their programme finished (OR = 2.07; CI = 1.20–3.57, *p* = 0.009).

#### Participants’ response to social support and group integration strategies

Social support, relatedness to others and feelings of belonging are all pillars of the MFL intervention programme. The process evaluation questionnaire at T1 and T2 assessed the perceptions of these variables (Supplementary Table S8). The intervention group were more likely than the usual provision group to receive support from other participants at both time points (T1: OR = 2.44, 95% CI = 1.31–4.56, *p* = 0.005; T2: OR = 3.38, 95% CI = 1.95–5.84, *p* = 0.001) and also to give support to other MFL participants at T2 to help them to become and remain active (OR = 2.87, 95% CI = 1.68–4.88, *p* = 0.001). The intervention group reported that they are more likely to remain active due to relationships they had formed (OR = 3.04, 95% CI = 1.65–5.60, *p* = 0.001); that they have developed new friendships as a result of MFL (OR = 3.36, 95% CI = 1.93–5.84, *p* = 0.001); and to report having kept in contact with participants from the MFL programme (OR = 2.24; 95% CI = 1.30–3.89; *p* = 0.004).

#### Interaction between participants and peer mentors

Intervention group participants were asked items about their views on peer mentors (Supplementary Table S9). A majority of participants were aware of (81.1%) and had been in contact with (81.2%) peer mentors. Almost three-quarters of participants found the peer mentors to be pretty helpful. Feedback from participants and the mentors themselves revealed several issues that need to be addressed to make best use of the role of peer mentors in the future. For example, in some of the interviews with participants it became apparent that there was confusion over who were the peer mentors in the group and their role. Seemingly, this confusion may have happened because mentors, and their role, were not always introduced to the participants:

“The group aren’t going to respond to the peer mentors unless they know that they are peer mentors and that they are actually…there for a purpose” (peer mentor).

#### Considerations about maintenance (sustainability) of the programme

From a sustainability perspective a focus of the MFL intervention was to strengthen the relationship between individuals with their LSP. The T2 process evaluation asked MFL participants questions to assess this level of engagement with the LSP on a 5-point Likert scale. The MFL intervention group were significantly more likely to be aware of their local LSP PA opportunities, and to have contacted their local LSP more frequently. In addition, engagement with their local Hub was significantly higher than either usual provision or the control group. While it is still a very small proportion of the entire sample who volunteer their services, the intervention group were eight times more likely to have volunteered to help run PA activities in their local Hub, and eight times more likely to have organised PA programmes at their Hub. Finally, more people in the MFL intervention group indicated that they met new people in their local community since they had joined MFL (see Table 3).

**Table 3:** Participant engagement with LSP since joining MFL.

	MFL intervention (*n* = 113)		Usual provision (*n* = 141)		Control (*n* = 90)	
	A great deal %	Rarely %	A great deal %	Rarely %	A great deal %	Rarely %
Become more aware of LSP PA opportunities (*p* = 0.009)**	52	48	40	60	30	70
Contacted my LSP about PA opportunities (*p* = 0.012)*	21	79	9	91	12	88
Registered for other LSP programmes	16	84	13	87	13	87
Volunteered to help run PA activities in my HUB (*p* = 0.007)*	8	92	1	99	1	99
Organised PA programmes at my HUB (*p* = 0.005)**	8	92	2	98	0	100
Attended PA in other venues (not my HUB)	34	66	27	73	30	70
I have met new people in my community (*p* = 0.008)**	36	64	27	73	17	73

Significant group difference at *p < 0.05 and **p < 0.005.

### Context of the Move for Life trial: how does context affect implementation

Aspects external to the intervention that may act as a barrier or facilitator to its implementation were considered (12). External barriers and facilitators related to venue and timing of sessions, seasonality and weather were identified as influencing implementation of PA programmes included in the MFL trial.

#### Venue and timing of sessions

Up to 87% (89% in intervention, 84% in usual provision) agreed that their venue was appropriate. In total, 79% (84% in intervention, 76% in usual provision) agreed that the timing of their sessions suited them. Although a high percentage of participants agreed their venue was suitable for the programme, some comments pointed to issues regarding lack of appeal of walking venues used and potential safety issues when walking on the road. Some instructors also commented about having to work around the challenge of non-ideal space for their programmes:

“The space was a little bit more restricted, but again you know you are not always given a great big sports hall to work in. You need to work around that as well. I’m kind of used to working around that as well with classes” (usual provision instructor).

Not having a venue also caused issues for completing the handbook as one instructor recalls:

“Depending on weather and things like that you were not always able to engage the participants with the book. For example, when we were walking and it rained one evening early in the summer, we could not, we had no venues, so we could not get everyone together outside to engage with the workbook” (intervention instructor).

Besides the venue, the timing of the session turned also to be important, either in a positive or negative way. For example, the Go for Life programmes were facilitated early in the day, allowing time for the recommended social cup of tea at the end of the class. Other programmes were scheduled at times that were not suitable for older adults, particularly for those with mobility issues. It was also noted that the timing of the sessions had an impact on participation:

“Not liking walking in the dark at (venue) as just had a hip replacement” (usual provision participant).

#### Seasonality and weather

It was also noted that the time of year when the programme runs is important, particularly for the programmes which operate outside, for example the cycling programmes:

“I think the timing of the course was good in the sense that it was the end of last year that [was] when it was run, but then in January, February, March when the weather is still bad and the people are not getting out on the road with bikes unless you are very into it” (usual provision instructor).

In this regard, one of the most cited barriers to participation related to the weather. For some who participated during the summer months, unusually hot weather made it difficult to attend some sessions, while holidays could also interfere with attendance:

“Was unable to attend some sessions due to extreme heat and some holidays” [intervention participant].

At the same time, those who participated in the winter noted similar problems related to cold weather.

“The bad weather which made me miss two sessions” [intervention participant].

### Costs

The methods employed for the cost analysis proved to be feasible and acceptable to participants. Table 4 presents the summary cost estimates at follow up by treatment arm. For the MFL intervention arm, the mean cost of organising, implementing and running the programme was estimated at €82.02 (SD: 41.01) per participant, and the mean healthcare cost over the 6 months follow up period was €272.70 (SD:1200.24), resulting in a mean total cost estimate of €354.71 (SD:1204.77) per participant. For the usual provision arm, the respective estimates were €43.61 (SD:48.67), €189.78 (SD:1064.90), and €233.39 (SD:1067.98). For the control arm, the mean total cost was estimated at €155.38 (SD: 436.07) per participant, which comprised solely healthcare resource costs over the 6 months.

**Table 4:** Cost estimates at follow up.

Variable/time point	Treatment arm					
	MFL intervention		Usual provision		Control	
Resource item	Mean	(SD)	Mean	(SD)	Mean	(SD)
GP visits	102	(162.92)	84	(86.32)	75	(72.60)
Practice nurse visits	22	(37.60)	18	(27.41)	17	(28.58)
Outpatient visits	47	(170.92)	24	(106.20)	37	(125.21)
Inpatient days	240	(1466.46)	239	(1563.66)	86	(441.58)
A&E visits	25	(100.59)	22	(128.05)	12	(56.77)
**Total healthcare cost**	€272.70	(1200.24)	€189.78	(1064.90)	€155.38	(436.07)
MFL intervention set up costs	36.97	(0.00)	0	(0.00)	0	(0.00)
Bike for life	81.67	(0.00)	81.67	(0.00)	0	(0.00)
Go for life	64.00	(0.00)	64.00	(0.00)	0	(0.00)
Get Ireland walking	40.00	(0.00)	40.00	(0.00)	0	(0.00)
Men on the move	202.00	(0.00)	202.00	(0.00)	0	(0.00)
Women on wheels	81.67	(0.00)	81.67	(0.00)	0	(0.00)
**Total programme cost**	€82.02	(41.01)	€43.61	(48.67)	€0.00	(0.00)
**Total cost**	€354.71	(1204.77)	€233.39	(1067.98)	€155.38	(436.07)

The results from the multilevel statistical analyses, which estimated incremental costs controlling for treatment arm, baseline costs and clustering, indicated that the MFL intervention was associated with a statistically insignificant increase in mean cost of €120 (95% CIs, −50.05, 289.25) relative to the usual provision arm, and a statistically significant increase in mean cost of €218 (95% CIs, 109.058, 326.746) relative to the control arm. Finally, the usual provision arm was associated with a statistically insignificant increase in mean cost of €78 (95% CIs, −102.065, 258.722) relative to the control arm.

The results from a series of sensitivity analyses for the MFL versus Usual Provision comparison are presented in Table 5 and generally support the findings from the base-case analysis. Notably, the exclusion of healthcare costs other than the implementation of the intervention had an important impact on the incremental cost estimates: €39 (95% CIs, 31.79, 45.65).

**Table 5:** Costing sensitivity analyses.

Analysis	Incremental analysis: MFL intervention vs. usual provision Difference in means (95% CI’s) [*p*-value]
*Base-case analysis*	€120 (95% CIs: −50.05, 289.25)
Cost of MFL intervention plus 10%	124 (−45.994, 293.179) [0.153]
Cost of MFL intervention minus 10%	116 (−54.114, 285.333) [0.182]
Cost of MFL intervention plus 50%	140 (−29.778, 308.912) [0.106]
Cost of MFL intervention minus 50%	100 (−70.378, 269.681) [0.251]
Univariate regression model estimated controlling for hub clustering	127 (−72.999, 326.296) [0.214]
MFL intervention cost only included (i.e., exclude other care costs)	39 (31.785, 45.649) [0.000]

CI, confidence intervals.

Multivariate regression estimated controlling for arm, baseline cost and hub clustering.

## Discussion

This study presents the process evaluation findings of the MFL trial. As per the Medical Research Council framework, the evaluation sought to expose links between implementation variables, mechanisms of impact and context of the intervention. As recommended in the RE-AIM framework, the evaluation also looked at the dimensions of sustainability and cost of the intervention. While in general the trial reached the target audience, recruitment of males and individuals from hard-to-reach groups proved challenging. The social aspect and the activities were frequently mentioned as the most enjoyable components of the intervention, while the weather, travel to distant venues, research burden and differences in ability levels among participants were mentioned as less enjoyed aspects. The evaluation highlighted several mechanisms potentially explaining the effects of the intervention, such as improved attitudes toward PA, more positive views about PA programmes and instructors, enriched interpersonal interaction, greater social support, and more frequent use of individual and group behaviour change techniques. From a sustainability perspective, the intervention demonstrated potential to strengthen the connection between individuals and their LSP. On an economic basis, the MFL intervention was associated with a statistically insignificant increase in mean cost compared to usual provision, and a statistically significant increase in mean cost relative to the control arm.

The data from the process evaluation help explain the promising results previously reported for the MFL intervention regarding outcomes related to energy expenditure (e.g., self-reported compliance with PA guidelines), body composition (waist circumference), physical function (e.g., functional mobility), psychosocial functioning (mental wellbeing, loneliness) and hypothesized mediators of PA (e.g., self-efficacy, subjective norms, attitudes, perceived relatedness to others) (7, 8). Notably, overall views and experience of the LSP PA programmes (e.g., finding their programme interesting, worth their time and enjoyable, being likely to recommend the programme to a friend) were significantly and considerably more positive among MFL participants than among usual provision participants at the end of the programmes. Likewise, the intervention group reported higher use of key behavioural change techniques (e.g., setting personal and group goals to increase physical activity, considering personal benefits and barriers to becoming more active) than the usual provision group, both at the end of the programmes and three-months later. Furthermore, compared to usual provision, more people in the MFL intervention group reported receiving support from other participants, having developed new friendships as a result of MFL, having kept in touch with participants from the MFL programme, having met new people in their local community since they joined MFL, and, importantly, that they are more likely to remain active due to relationships they have formed. While the primary outcomes for MFL concerned energy expenditure, MFL also addressed the increasing problem of social isolation (26, 27) through its core themes of social support, group integration and cohesion, and related intervention strategies (6, 8). Fittingly, as illustrated in the results, the most valuable aspect of the MFL programme according to the participants was the social interaction, often in combination with features of the programme activities. Moreover, instructors in the MFL intervention arm received significantly higher ratings than usual provision on mixed ability teaching and providing participants with more choices on how to perform the activities, despite instructors in the usual provision arm being very highly rated by participants. Substantial increases in confidence scores pre- and post- MFL instructor training show the value of the training provided to instructors to deliver behaviour change strategies within their PA programmes and may explain the higher ratings that MFL instructors received from participants compared to instructors in the usual provision arm.

In the MFL group, increasing age was significantly and positively related to variables such as appropriate timing of the programme and amount of information provided, learning about new strategies to keep physically active, goal setting, and providing and receiving social support from peers. Insufficient PA increased from 2000 to 2022 in people aged 60 years and older in all regions of the world, regardless of gender (28). Thus, process evaluation findings from the MFL trial are encouraging considering current trends, since it is important that PA promotion efforts do not exacerbate existing age-related inequalities by, for example, ‘age-proofing’ the design of PA interventions for older adults (4, 28). Nevertheless, although the MFL trial was successful in recruiting, and catering to, inactive older adults, the findings also indicate that recruitment strategies need to be refined to reach males and individuals from hard-to-reach groups based on socioeconomic characteristics, an issue that has been previously reported in other PA interventions for adults and older adults (29).

In addition to focus on cost and resources needed for the intervention, the RE-AIM framework directed our attention to information that may be indicative of potential for maintenance or sustainability of the MFL intervention (13). From this perspective a focus of the MFL intervention was to strengthen the relationship between individuals with their LSP. This was envisioned as activating individuals who would become a community of practice and invigorate their local sport and PA Hub. For this to happen, participants would need, for example, to become more aware of opportunities available in their local community, making contact more frequently with the LSP and/or volunteering or organising events, which was supported overall in the results reported in the corresponding section.

A refined programme theory/logic is one of the most important outcomes of an evaluation, particularly where a theory-based perspective is adopted (30). While the process evaluation, particularly regarding the analysis in the mechanisms of impact domain of the MRC framework, supports the overall suitability of the original MFL logic model (6), it also suggests three major areas for improvement in the current MFL components.

First, use of the participant handbook for the delivery of cognitive-behavioural skills (e.g., problem solving, goal setting) needs to be reconsidered. One possibility could be to integrate the current content in the handbook in a more fluid manner with other MFL content and regular PA programming via use of motivational interviewing techniques by instructors (31). Another possibility, which could complement the previous, particularly in the context of post pandemic increase in use of online resources and platforms (32), would be to convert the participant handbook to an app, both for practical and sustainability purposes. This could help address concerns that were raised about the practicality of using the handbook due to the nature of the activity (e.g., cycling) and issues about participant engagement with the weekly ‘homework tasks’, as well as the extent to which the handbook is followed by the instructors as intended. Likewise, this could make the handbook more educational with greater focus on exercises and stretches with pictures and demonstrations of how these could be done at home. Although this was not a focus of MFL, it was something that the participants suggested and should be considered for further development.

Second, the number of MFL behaviour change strategies currently included in the programme could be reduced, particularly in 8- and 10-week programme formats, to incorporate only those that were more consistently implemented by instructors and acceptable to participants. Third, there is a need for better integration of training of peer mentors and instructors, so the potential of the former within the MFL programme is maximised. To this aim, we recommend that instructors attend part of the peer mentor training. Although strategies for embedding the peer mentor within the PA programmes were discussed during training of instructors, these need to be more explicitly covered in practice. This would help the instructors to integrate the peer mentors in the group and use their new skills, and at the same time foster recognition of the peer mentor role and positive endorsements by the groups.

The cost analysis confirmed the feasibility of the methods employed and provided micro-costed estimates for the costs of implementing the strategies to encourage physical activity among middle-to-older aged adults living in the community in Ireland. These estimates will be useful for future studies seeking to definitively assess the cost effectiveness of such programmes. To this end, incremental estimates for costs and health outcomes will need to be jointly considered in the context of the €20,000 to €45,000 per QALY threshold value range that is employed to inform decision-making in Ireland (23). Future health economic evaluation studies should consider employing definitive randomised trial designs and decision modelling techniques to extend the time horizon of analysis to capture costs and health outcomes over the lifetime of participants. Further, they should consider the inclusion of a wider set of health and community care resource categories, productivity losses, and private out-of-pocket expenses.

### Strengths and limitations

Adhering to a recognised process evaluation framework addresses the need for clarity in reporting and integration of findings. The evaluation’s mixed methods design integrating quantitative and qualitative phases, results, and data facilitated the incorporation of views from diverse stakeholders and the understanding of the mechanisms that drive intervention outcomes. Similarly, considering key stakeholders in the cost analysis will support appropriate decision making about resource allocation accounting for different, complementary perspectives, namely the sport sector service provider (LSPs) and the publicly funded health and social care system in Ireland (the Health Service Executive). Conversely, we were limited by resources and time regarding the number of perspectives that we could incorporate both in the process evaluation and the cost analysis, which was not exhaustive in the first case and did not represent a full societal perspective in the second (33). Another limitation concerns the assessment of some variables in this study using validated measures. While the mediation analysis performed lends some support to the hypothesized psychosocial determinants of change in PA behaviour (particularly attitudes toward physical activity), ceiling effects in the measures we used to assess these determinants may have affected our ability to detect meaningful differences across time in this analysis. Lastly, although we report on the process evaluation of a cluster randomised feasibility trial, the representativeness of the findings needs to be interpreted in light of the characteristics of the sample. In particular, recruitment of men was difficult. Gender sensitised recruitment strategies were used by MFL, but completion of the ‘health-checks’ was open to both males and females. This may have prevented some men from attending and needs to be considered for future optimal recruitment.

## Conclusion

This study presents the process evaluation findings of the MFL trial for inactive adults aged 50 years and older. The evaluation was guided by MRC framework, complemented with selected elements from the RE-AIM framework. The process evaluation sheds light on the mechanisms underlying the promising results previously reported for the MFL intervention regarding outcomes related to energy expenditure, body composition, physical function, psychosocial functioning and hypothesized mediators of physical activity. Inclusion of key stakeholders in the cost analysis will enable appropriate decision making about resource allocation considering relevant perspectives on the intervention. While the process evaluation supports generally the original MFL intervention logic model, it also suggests several areas for improvement in the current MFL components, including reconsidering the use of the participant handbook for the delivery of behavioural skills as well as the number of behavioural change strategies, particularly in shorter duration programme formats, and better integrating the training of peer mentors and PA programme instructors.

## Data Availability

The datasets presented in this article are not readily available because the raw data supporting the conclusions of this article will be made available by the authors upon reasonable request. Requests to access the datasets should be directed to catherine.woods@ul.ie.
