# Supplementary material for: Process evaluation with cost analysis of the Move for Life cluster randomised feasibility trial for inactive adults aged 50 years and older
Source: Front Public Health. 2025 Dec 5;13:1681089. doi: 10.3389/fpubh.2025.1681089 (PMC12714609; doi:10.3389/fpubh.2025.1681089)
Supplement: Supplementary file 1 [file Table_1.docx]

| **Participant Interview Guide**  **Icebreaker**  **Background**  What did you think of the MFL [specific programme name] programme?  Why did you keep coming to the programme  What worked well in the programme?  Is there anything else you would have wanted included in the programme?  **Programme Factors**  What did you think of the tutors and the way they delivered the programme?  What did you think of the group?  What did you think of the peer mentors?  What did you think of the handbook used in your activity?  **Outcomes of the programme**  Which parts of Move for Life were most valuable to you?  Which parts of Move for Life were least valuable to you?  What are the main differences being on the programme has made to you?  Did being on the programme have any effect on your family and friends?  **Sustainability**  Do you plan to continue following the Move for Life programme?  Are any of you involved in continuing in helping your hub to continue the physical activity?  If we were to run the MFL programme again, in a different HUB what advice would you have for us to continue this programme?  **Closing (5 mins)**  We are now approaching the end of our discussion. Is there anything else anyone would like to add about the Move for Life programme we have not talked about? |
| --- |

| **Peer Mentor Interview Guide**  **Icebreaker**  **Background (Demographics)**  Is this your first physical activity programme?  How would you describe yourself?  **MFL procedure**  Tell me how you became a peer mentor?  What made you interested in becoming a peer mentor?  Can you tell me some specifics about the Move for Life programme (specific name) as it was delivered in (specific place)?  **Training Factors**  Tell me about your experience with the peer mentor training?  Is there any aspect of the training that you needed more support with?  Tell me about how you felt about first applying the peer mentor training with the group?  **Programme Factors**  How do you feel Move for Life (specific programme) went?  If you were to explain the MFL intervention to a friend, how would you describe it?  From your perspective, what was move for life trying to achieve?  Describe how you aided the tutor with the MFL programme?  Tell me about how you interacted with the group.  Can you tell me about a time when you used a strategy taught to you in the training with the group?  On a scale of 1 to 10, with 1 ease of delivery being unfriendly and 10 being very user friendly, where would you place MFL?  On a scale of 1-10 with 1 being not relevant to getting inactive people more active and 10 relevant, how would you rate MFL?    **Specific Elements of MFL: Workbook**.  Did you follow the workbook in the programme?  Out of the strategies that were provided for you from MFL training and workbook, which were the MOST useful?  Out of the strategies that were provided for you from MFL training and workbook, which were the least useful?  What strategies did you use in your programmes from the training and workbook?  What strategies did you not use?  Tell me about how you encouraged the group to engage with the homework.  Describe for me how you felt being a peer mentor within your group?  Describe how you found interacting with the group?    **Peer Mentors**  In your opinion, what is the value of a peer mentor in a programme like Move for Life?  Would this work for other programmes?  Describe for me your role as a Move for Life peer mentor.  **Outcomes of the programme**  Do you think Move for Life was valuable for increasing physical activity?  Which part of the programme was most useful?  Did you notice any unexpected or unanticipated consequences (positive or negative) for the participant, the partnership or for yourself from delivering Move for Life?  **Barriers and facilitators**  What helped you be a peer mentor for Move for Life?  What hindered your delivery of Move for Life?  **Sustainability**  Tell me about your plans for the group now that the programme has finished?  What would help the partnership to continue the programme?  Do you have any suggestions about how we might improve MFL for the tutors, the participants and for the partnership?  **Closing**  We are now approaching the end of our discussion. Is there anything else anyone would like to add about the Move for Life programme we have not talked about? |
| --- |

| **Tutor (Instructor) Interview Guide**  **Icebreaker**  **Background (Demographics)**  What is your background in relation to coaching?  **MFL procedure**  How did you get involved in delivering the programme?  What made you interested in delivering Move for Life (specific programme)?  Can you tell me some specifics about the Move for Life programme (specific name) as it was delivered in (specific place)?  Did you have any issues with participants turning up for classes?  **Training Factors**  How did you find the training? (Training with Enrique, Catherine and Nollaig)  Was there anything too simple?  **Programme Factors**  How do you feel Move for Life (specific programme) went?  If you were to explain the MFL intervention to another tutor in another LSP, how would you describe it?  From your perspective, what was move for life trying to achieve?  On a scale of 1 to 10, with 1 ease of delivery being unfriendly and 10 being very user friendly, where would you place MFL?  On a scale of 1-10 with 1 being very difficult to apply and 10 being easy how would you rate MFL?  On a scale of 1-10 with 1 being not interesting to 10 being very interesting how would you rate MFL?  On a scale of 1-10 with 1 being not relevant to getting inactives more active and 10 relevant, how would you rate MFL?  **Specific Elements of MFL: Workbook.**  Did you follow the workbook in the programme?  Out of the strategies that were provided for you from MFL training and workbook, which were the most useful?  Out of the strategies that were provided for you from MFL training and workbook, which were the least useful?  What strategies did you use in your programmes from the training and workbook?  What strategies did you not use?  Did you find the homework a useful tool?  Did you engage in group discussions to cover the weekly content in the workbook?  Do you think the participants engaged with the workbook/homework/discussions?    **Peer Mentors (for intervention tutors only)**  Did you recruit PM?  In your opinion, what is the value of a peer mentor in a programme like Move for Life?  Would this work for other programmes? (Sustain physical activity)    **Outcomes of the programme**  Do you think Move for Life was valuable for increasing physical activity?  Which part of the programme was most useful?  Did you notice any unexpected or unanticipated consequences (positive or negative) for the participant, the partnership or for yourself from delivering Move for Life?  **Barriers and facilitators**  What helped you to deliver Move for Life?  What hindered your delivery of Move for Life?  Do you feel MFL is compatible with your work at the partnership?  Do you think a community of learning between other SDO’s would work?    **Sustainability**  If the partnership keeps using Move for Life, will you be involved?  What would help the partnership to continue the programme?  Do you have any suggestions about how we might improve MFL for the tutors, the participants and for the partnership?  **Closing**  We are now approaching the end of our discussion. Is there anything else anyone would like to add about the Move for Life programme we have not talked about? |
| --- |

| **MFL Facilitator Interview Guide**  **Background**  What is your background in relation to physical activity and health?  **Role in Move for Life**  Can you tell me about your role in Move for Life?  **The Intervention**  Can you describe the MFL intervention for me?  Can you detail the timeline of MFL?  **The tutors**  Can you tell me about the role of the MFL tutor  Can you tell me about the training workshop for tutors?  Tell me about how you worked with them after training workshop (WhatsApp etc.)?  Tell me about your main learnings of tutor involvement in MFL?  Based on your experience, what makes a good tutor on MFL?  What additional supports may be required for tutors?  **The Peer Mentors**  Can you tell me about the role of the peer mentor?  Can you tell me about the training workshop for peer mentors  Tell me about how you worked with them after training workshop (WhatsApp etc.)?  Tell me about your main learnings of peer mentor involvement in MFL?  What makes a good peer mentor on MFL?  What additional supports may be required for peer mentors?  **The Resources**  Can you tell me about the manual for tutors?  Can you tell me about the WhatsApp group for tutors?  **Evaluation of Experience**  Can you tell me the strength of LSP involvement in MFL?  Can you tell me opportunity to improve LSP involvement?  Can you tell me the strength of tutor involvement in MFL?  Can you tell me opportunity to improve tutor involvement?  Can you tell me the strength of peer mentor involvement in MFL?  Can you tell me opportunity to improve peer mentor involvement?  Can you tell me the strength of UL involvement in MFL?  Can you tell me opportunity to improve UL involvement?  Overall, If we were to do it all again what would your top tips be? |
| --- |
