# Supplementary material for: Process evaluation with cost analysis of the Move for Life cluster randomised feasibility trial for inactive adults aged 50 years and older
Source: Front Public Health. 2025 Dec 5;13:1681089. doi: 10.3389/fpubh.2025.1681089 (PMC12714609; doi:10.3389/fpubh.2025.1681089)
Supplement: Supplementary file 2 [file Table_2.DOCX]

Table SM1. Comparison between individuals included and excluded from MFL study

|  | Included in MFL  N=601 | Excluded from MFL  N=132 |  |
| --- | --- | --- | --- |
| Gender (%)  Male  Female | 19.6%  80.4% | 29.9%  70.1% | p<.001^1^ |
|  | Mean (SD) | | |
| Age (years) | 63.06 (8.1) | 59.40 (9.8) | p<.001^2^ |
| #Days.30mins.MVPA^3^ | 2.61 (1.8) | 4.71 (2.1) | p<.001^2^ |
| Average MVPA per day (in bouts of 10 minutes)^4^ | 13.02 (14.3) | 32.12 (30.2) | p<.001^2^ |

*Note*. ^1^ Crosstabs with chi square; ^2^Independent samples t-tests, ^3^Measured by M-1 self-report instrument, ^4^Measured by activPAL accelerometer

Table SM2. Characteristics of MFL study participants in comparison to HaPAI data %

|  | HaPAI Limerick City | HaPAI Limerick County | MFL Limerick | HaPAI Clare County | MFL Clare |
| --- | --- | --- | --- | --- | --- |
| *Employment status* |  |  |  |  |  |
| At work | 21 | 30 | 36 | 29 | 42 |
| Unemployed | 5.9 | 4.9 | 3.7 | 5.3 | 3.8 |
| Student | 0.3 | 0.2 | .9 | 0.2 | 0.8 |
| Unable to work due to illness/disability | 11 | 7.5 | 7.1 | 6.7 | 2.9 |
| Retired | 48 | 43 | 44 | 44 | 41 |
| Look after home/family | 13 | 15 | 7.1 | 14 | 7.1 |
| Other | 0.4 | 0.3 | 0.6 | 0.4 | 1.7 |
| *Educational attainment* |  |  |  |  |  |
| Primary or no formal training | 32 | 31 | 7.2 | 7 | 8.0 |
| Lower secondary | 18 | 20 | 16 | 40 | 14 |
| Upper secondary | 23 | 26 | 25 | 20 | 21 |
| Post-secondary non-tertiary | - | - | 3.4 | - | 6.0 |
| Non-degree | 2.5 | 3 | 26 | 3 | 23 |
| Degree or higher | 10 | 11 | 22 | 12 | 28 |
| *Marital status* |  |  |  |  |  |
| Married/living with partner | 58 | 64 | 67 | 63 | 63 |
| Widowed | 19 | 17 | 16 | 17 | 13 |
| Separated/divorced | 9.1 | 5.6 | 10 | 7 | 12 |
| Single/never married | 14 | 14 | 7 | 13 | 12 |

Table SM3. Reasons for dropout of study n (%)

|  | Baseline (T0) | Follow up (T1) | Follow up (T2) |
| --- | --- | --- | --- |
| No reason | - | 47 (34.8) | 53 (81.5) |
| Other commitments | - | 5 (3.7) | 5 (7.7) |
| Personal reasons | - | 3 (2.2) | - |
| Medical reasons | - | 8 (5.9) | 1 (1.5) |
| Didn’t like program | 1 (0.2) | 3 (2.2) | 1 (1.5) |
| Lack of time | - | 1 (0.7) | 1 (1.5) |
| Didn’t receive program | - | 64 (47.4) | 1 (1.5) |
| Family commitments | - | 4 (3.0) | 3 (4.6) |
|  | - |  |  |
| Total | 1 (0.2) | 135 (25.8) | 64 (12.2) |

Table SM4. Participant overall views and experience of the LSP PA programme

|  | Intervention  (n=121) | | Usual Provision (n=141) | | p-value |
| --- | --- | --- | --- | --- | --- |
|  | A  % | D  % | A  % | D  % |  |
| T1: The MFL programme was relevant | 96 | 4 | 91 | 9 | p>.05 |
| T1: The MFL programme was interesting | 96 | 4 | 90 | 11 | p<.05 |
| T1: MFL was worth the time I invested into the programme | 96 | 4 | 87 | 13 | p<.05 |
| T1: Overall, I enjoyed the Move for Life programme | 98 | 2 | 92 | 8 | p>.05 |
| T1: Overall, my group enjoyed the MFL programme | 91 | 9 | 82 | 18 | p<.05 |
| T1: I would recommend MFL to a friend | 96 | 4 | 89 | 11 | p<.05 |
| T2: The ‘Health check’ assessment motivated me to keep active | 43 | 57 | 98 | 2 | p<.05 |

*Note*. A= agree, D = disagree

Table SM5. Participant views on instructors

|  | Intervention  Group  (N=121) | | Usual Provision  (N=141) | | p-value |
| --- | --- | --- | --- | --- | --- |
|  | A  % | D  % | A  % | D  % |  |
| T1: The tutors were knowledgeable about physical activity and health | 98 | 2 | 93 | 7 | p>.05 |
| T1: The tutors provided clear information about the activities | 96 | 4 | 95 | 5 | p>.05 |
| T1: The tutors gave me useful feedback on how I could improve^1^ | 81 | 19 | 71 | 28 | p=.05 |
| T1: The tutors provided me with choices on how to perform the activities | 87 | 13 | 72 | 28 | p<.01 |
| T1: The tutors encouraged me to work at my own pace | 95 | 5 | 90 | 10 | p>.05 |
| T1: The tutors offered a wide variety of activities to meet the ability of all people within the group | 85 | 15 | 76 | 24 | p<.05 |
| T1: The tutors were approachable and friendly | 99 | 1 | 97 | 3 | p>.05 |
| T1: The tutors were enthusiastic and motivating | 96 | 4 | 94 | 6 | p>.05 |
| T1: The tutors were organised and well prepared | 96 | 4 | 94 | 6 | p>.05 |
| T1: The tutors made me feel part of the group | 94 | 6 | 92 | 8 | p>.05 |
| T2: The tutors encouraged our group to continue to be active together after the programme had finished | 96 | 4 | 80 | 20 | p<.01 |

*Note*. A=agree, D = disagree.

Table SM6. Participant views on MFL handbook and materials

|  | **Total (n=118)** | |
| --- | --- | --- |
|  | **A (%)** | **D (%)** |
| T1: The workbook for MFL was appealing | 78 | 22 |
| T1: I engaged with the 'homework tasks' every week | 56 | 44 |
| T1: The weekly homework tasks were easy to understand | 80 | 20 |
| T1: The weekly homework tasks were useful | 68 | 32 |
| T2: I read my MFL handbook | 70 | 30 |

*Note*. A=agree; D = disagree.

Table SM7. Behaviour change strategy use within the MFL trial physical activity programmes

|  | Intervention  (n=121) | | Usual Provision (n=141) | | p-value |
| --- | --- | --- | --- | --- | --- |
|  | A  % | D  % | A  % | D  % |  |
| T1: The amount of information each week was appropriate | 82.1 | 17.9 | 78.2 | 21.8 | p>.05 |
| T1: I enjoyed learning about a variety of strategies to keep physically active | 88 | 12 | 76 | 24 | p<0.01 |
| T1: I learned how to set personal goals to increase my physical activity | 84 | 16 | 66 | 34 | p<0.01 |
| T1: Our group set a clear 'group goal' to help keep us physically active | 73 | 27 | 51 | 49 | p<0.01 |
| T2: I have found MFL useful for helping me to remain active | 80 | 20 | 71 | 29 | p>.05 |
| T2: I have considered the personal benefits of becoming more active | 93 | 7 | 85 | 15 | p<.05 |
| T2: I have discussed the personal barriers to becoming more active | 85 | 15 | 74 | 26 | p<.05 |
| T2: I have used the MFL strategies to help me overcome setbacks | 94 | 6 | 96 | 4 | p>.05 |
| T2: I have thought about what motivates met to change my lifestyle. | 69 | 31 | 76 | 25 | p>.05 |
| T2: During MFL, our group set a clear 'group goal' to help keep us physically active after the programme finished | 73 | 27 | 57 | 43 | p<.05 |

*Note*. A= agree, D = disagree.

Table SM8. Social support within MFL intervention and usual provision groups

|  | Intervention  (n=118) | | Usual Provision (n=141) | | p-value |
| --- | --- | --- | --- | --- | --- |
|  | A  % | D  % | A  % | D  % |  |
| I have received support from other MFL participants that helped me be physically active  T1  T2 | 84  67 | 16  33 | 70  42 | 30  58 | p<0.01  p<0.01 |
| I gave support to other MFL participants during the programme that helped them to become physically active  T1  T2 | 63  61 | 37  39 | 55  39 | 45  61 | p>.05  p<0.01 |
| I am more likely to remain physically active because of the relationship I have formed with my group  T1 | 82 | 18 | 64 | 36 | p<.05 |
| I have developed new friendships as a result of being part of the MFL programme  T2 | 75 | 25 | 49 | 51 | p<0.01 |
| I have remained physically active during the last 3 months because of the relationship I formed with my MFL group  T2 | 87 | 13 | 89 | 11 | p>.05 |

*Note*. A= agree, D = disagree

Table SM9. Participant views on peer mentors

|  | **Intervention**  **(N=117)**  **N (%)** | |
| --- | --- | --- |
|  | **A (%)** | **D (%)** |
| T1: I was aware of the 'peer mentors' in my programme | 80 | 20 |
| T1: I found the 'peer mentors' pretty helpful | 74 | 26 |
| T1: I enjoyed the MFL programme more because of the 'peer mentors | 70 | 30 |
| T1: I engaged with the 'peer mentors' when I needed clarity on the MFL handbook | 60 | 40 |
| T2: I have been in contact with the peer mentors who were in my group? (since the MFL programme ended) | 73 | 27 |

*Note*. A=agree, D = disagree
