# Supplementary material for: Process evaluation with cost analysis of the Move for Life cluster randomised feasibility trial for inactive adults aged 50 years and older
Source: Front Public Health. 2025 Dec 5;13:1681089. doi: 10.3389/fpubh.2025.1681089 (PMC12714609; doi:10.3389/fpubh.2025.1681089)
Supplement: Supplementary file 3 [file Table_3.DOCX]

**
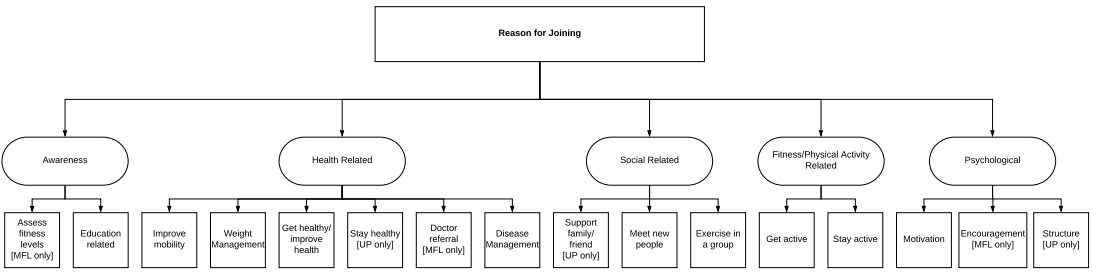
**

Figure 1. Reasons for joining Move for Life


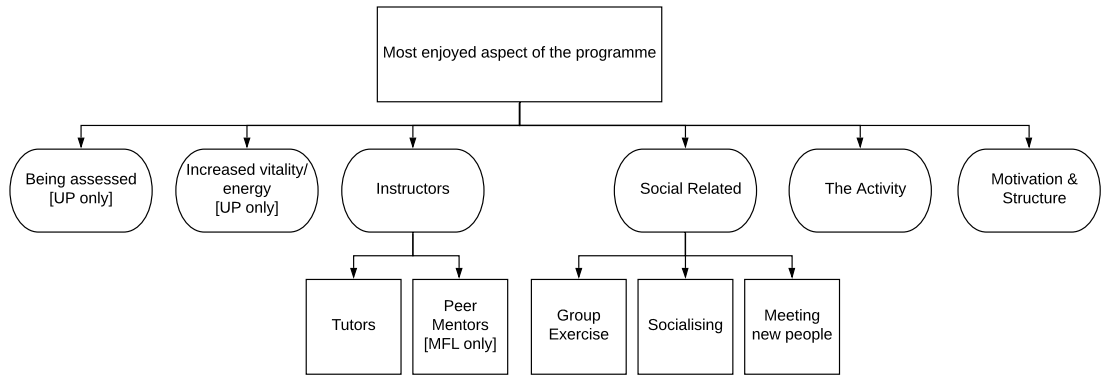


Figure 2. Most enjoyed aspect of the programme


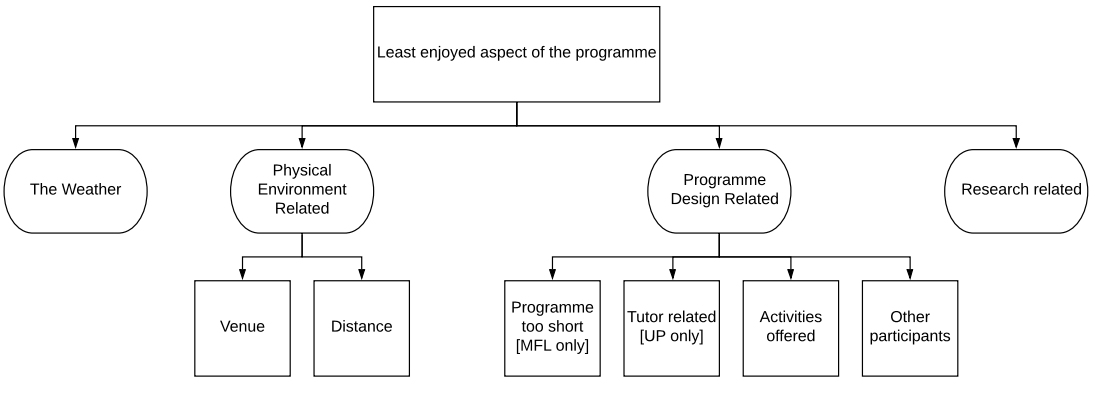


Figure 3. Least enjoyed aspect of the programme


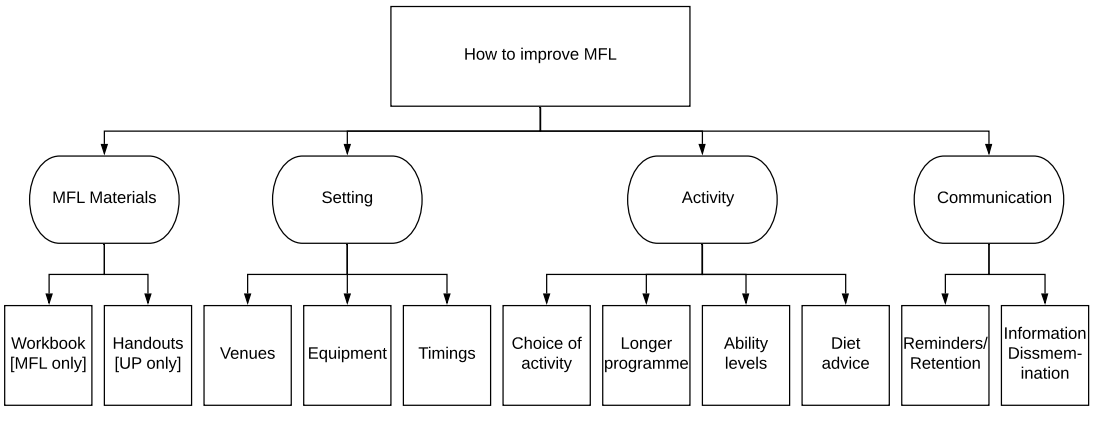


Figure 4. How to improve the programme
